# Secondary task performance during challenging walking tasks and freezing episodes in Parkinson’s disease

**DOI:** 10.1007/s00702-016-1516-7

**Published:** 2016-03-31

**Authors:** Valeria Dibilio, Claudia Stummer, Linda Drenthen, Bastiaan R. Bloem, Jorik Nonnekes, Vivian Weerdesteyn

**Affiliations:** Department of Rehabilitation, Radboud University Medical Centre, Donders Institute for Brain, Cognition and Behaviour, Nijmegen, The Netherlands; Department GF Ingrassia, Section of Neurosciences, University of Catania, Catania, Italy; Department of Neurology, Radboud University Medical Centre, Donders Institute for Brain, Cognition and Behaviour, Nijmegen, The Netherlands; Sint Maartenskliniek Research, Development and Education, Nijmegen, The Netherlands

**Keywords:** Parkinson’s disease, Gait disorders, Freezing of gait, Executive functions

## Abstract

Parkinson’s disease (PD) patients likely use attentional strategies to compensate for their gait deficits, which increases the cognitive challenge of walking. The interplay between cognitive functions and gait can be investigated by evaluating the subject’s attendance to a secondary task during walking. We hypothesized that the ability to attend to a secondary task decreases during challenging walking conditions in PD, particularly during freezing of gait (FOG)-episodes. Twenty-nine PD patients and 14 age-matched controls performed a simple reaction task that involved squeezing a ball as fast as possible in response to an auditory stimulus. Participants performed this reaction task during four conditions: (1) walking at preferred speed; (2) walking with short steps at preferred speed; (3) walking with short steps, as rapidly as possible; (4) making rapid full turns. We used surface electromyography to determine reaction times, and a pressure sensor located within the ball to determine movement onset. Reaction times of PD patients were slower (on average by 42 ms) compared to controls, regardless of the walking task. In both groups, reaction times were significantly longer during the turning condition compared to all other conditions. FOG-episodes were most often seen during the turning condition. In PD patients, reaction times were significantly longer during FOG-episodes compared to trials without FOG. Our results suggest that turning requires more attentional resources than other walking tasks. The observation of delayed reaction times during FOG-episodes compared to trials without FOG suggests that freezers use additional resources to overcome their FOG-episodes.

## Introduction

Gait deficits are common and debilitating signs of Parkinson’s disease (PD). Gait impairments in PD include reduced speed and stride length, and increased stride-to-stride variability (Yogev-Seligmann et al. 2012; Fasano et al. 2013). In the more advanced stages, festination and freezing of gait (FOG) can emerge (Morris et al. 2001; Kelly et al. 2012). FOG is a gait disorder characterized by sudden, relatively brief episodes of inability to step, or by extremely short steps (Nutt et al. 2011; Nonnekes et al. 2015). FOG is frequently evoked by challenging walking tasks such as walking with short steps or by turning as rapidly as possible (Chee et al. 2009; Snijders et al. 2012; Spildooren et al. 2010). It has been hypothesized that PD patients increase their attention during walking to compensate for their gait deficits (Yogev-Seligmann et al. 2012; Yogev et al. 2005; Rochester et al. 2014). When cognitive compensation becomes insufficient, particularly when challenging walking tasks further increase attentional and executive demands, FOG might emerge (Giladi et al. 2006; Vandenbossche et al. 2013).

The interplay between cognitive functions and gait can be investigated by evaluating a secondary task during gait, as it creates competition for attention and allocation of cognitive resources (Yogev-Seligmann et al. 2012; Woollacott et al. 2002). Indeed, during turning (as an example of a complex gait task), performance on a cognitive secondary task is poorer as compared to normal walking, and most evidently so in PD patients with freezing of gait (Yogev et al. 2005). However, it is unknown whether the greater decrements in secondary task performance in PD patients are also seen during other walking tasks that frequently evoke FOG, such as walking with small rapid steps (Chee et al. 2009; Snijders et al. 2012). If so, these results would further support the hypothesis that FOG may occur as a manifestation of insufficient attentional compensation during challenging gait tasks. Furthermore, it is unknown whether the poorer secondary task performance during turning—as observed previously in the freezers—was indeed related to the greater attentional resources needed for executing this rather difficult motor task, or alternatively, resulted from the utilization of additional attentional resources to overcome FOG-episodes evoked by the turning task.

Here, we hypothesized that the ability to attend to a secondary task disproportionally decreases during challenging walking conditions in PD, and particularly during FOG-episodes. To test this idea, we evaluated manual reaction times (in response to an auditory stimulus) in PD patients and healthy controls during walking, walking with short steps, walking with short steps as rapidly as possible and full rapid turns in both directions, both during FOG-episodes (if present) and during normal task execution.

## Materials and methods

### Participants

Twenty-nine patients with PD participated. All patients were diagnosed according to the UK Brain Bank criteria (Hughes et al. 1992). Exclusion criteria were any other neurological or orthopedic disorder affecting gait, severe cognitive impairment and medication negatively affecting gait or balance. All PD patients were measured in an OFF-state, when they experienced an end-of-dose effect prior to intake of their next medication dose. In addition, 14 healthy controls of similar age were included. The study was approved by the local medical ethics committee and was conducted in accordance with the Declaration of Helsinki and with local ethical guidelines. All subjects gave their written informed consent prior to the experiment.

### Clinical assessment

PD patients were assessed clinically with the motor subsection (part III) of the MDS-Unified Parkinson’s Disease Rating Scale (UPDRS, score/132) (Goetz et al. 2008). Patients also completed the New Freezing of Gait Questionnaire (N-FOGQ, score/33) (Nieuwboer et al. 2009). Global executive function was tested using the Frontal Assessment Battery (FAB, score/18) (Dubois et al. 2000).

### Experimental set-up and protocol

Participants performed a manual simple reaction time task under four conditions of increasing complexity; (1) while walking at preferred speed; (2) while walking with short steps (approximately 25 % of step length) at preferred speed; (3) while walking with short steps as rapidly as possible; (4) while making rapid axial 360° turns in both directions. The gait tasks were performed on a 4-m walkway. The order of the conditions was counterbalanced across subjects.

The simple reaction time task involved squeezing a rubber ball (6 cm in diameter) as fast as possible in response to an auditory stimulus (50 ms of white noise at 70 dB sound pressure level). The participants were instructed continue walking or turning when they heard the stimulus. PD patients held the ball in their most affected hand and controls in their dominant hand. The stimulus was generated by a custom made noise generator, and delivered through binaural earphones (Sennheiser, type HD518). The experimenter (VD) administered the stimulus via a button press on a keyboard that was concealed to the participants. The protocol included twelve repetitions of each gait task, each involving one auditory stimulus delivered at unpredictable moments. The experimenter, who was experienced in recognizing FOG-episodes, aimed to administer stimuli during both non-freezing and freezing episodes (if present). Prior to each task, subjects were allowed a few practice trials.

### Data collection

Electromyographic (EMG) data were collected from the flexor digitorum muscle and extensor carpi radialis (ZeroWire, Aurion, Italy). Self-adhesive Ag–AgCl electrodes (Tyco Arbo ECG) were placed approximately 2 cm apart and longitudinally on the belly of each muscle, according to Seniam guidelines (Hermens et al. 1999). Furthermore, to assess movement onset, a wireless pressure sensor (ZeroWire, Aurion, Italy) was placed inside the ball. Both EMG and sensor signals were sampled at 2000 Hz. Each trial was videotaped for 4 s following administration of the auditory stimulus by two cameras in the frontal and sagittal plane of the walkway to verify the presence or absence of FOG.

### Data analysis

#### Reaction time parameters

Two reaction time parameters were assessed: EMG onset latencies (from flexor digitorum and extensor carpi radialis) and pressure-sensor onset latency. First, EMG data were full-wave rectified and low-pass filtered at 30 Hz (zero-lag, second order Butterworth filter). Muscle onset latencies were determined using a semi-automatic computer algorithm that selected the first instant at which the EMG activity exceeded a threshold of 2 SD above the background activity, as calculated over a 500 ms period just prior to the auditory stimulus. Onsets were first selected by the computer algorithm, then visually approved and (when necessary) corrected. Onset latencies were determined for each trial separately. Movement onset as recorded from the pressure sensor inside the ball was determined in the same manner.

#### FOG-episodes

Two independent and experienced raters (VD and CS) scored the videos for the presence of FOG.

### Statistical analysis

We first tested for differences in reaction times between PD patients and controls, discarding reaction times during FOG-episodes. We used a repeated measures ANOVA, with task (normal walking–walking with short steps–walking with short steps rapidly-turning) as within-subject factors and group (PD patients-controls) as between-subjects factor. In case of a significant task effect, we used post hoc paired *t* tests to identify differences in reaction times between tasks. Finally, for patients who showed freezing during the measurement, we compared outcome measures during FOG-episodes and during trials without FOG using a paired samples *t* tests.

## Results

### Clinical assessment

Clinical characteristics of the participants are shown in Table 1. PD patients and controls did not differ with respect to age [*t*(41) = 1.025, *p* = 0.311] or gender (*χ*^2^ = 0.1, *p* = 0.75).

**Table 1:** Participants characteristics

	PD patients (*n* = 29)	Controls (*n* = 14)
Age (years)	65.3 (48–83)	67.7 (58–74)
Gender	22 M	10 M
Disease duration (years)	9 (2–21)	
Hoehn and Yahr stage		
2	21	
2.5	4	
3	4	
MDS-UPDRS III	37.5 (18–59)	
UPDRS-PIGD items	3.8 (0–8)	
UPDRS-bradykinesia items	17.4 (6–27)	
N-FOGQ	9.1 (0–23)	
FAB	16.6 (13–18)	

Data represent mean (range) and frequency. For both MDS-UPDRS and N-FOGQ, higher scores indicate worse functioning. For FAB, lower scores indicate worse functioning

*MDS- UPDRS III* MDS-Unified Parkinson’s Disease Rating Scale part III, *PIGD*-*items* postural instability/gait difficulty items (item 9–13; score/20), bradykinesia items (item 4–8 and 14; score/44), *N*-*FOGQ* New Freezing of Gait Questionnaire (score/33), *FAB* Frontal Assessment Battery (score/18)

### Reaction times in PD patients versus control subjects

**Fig. 1:**
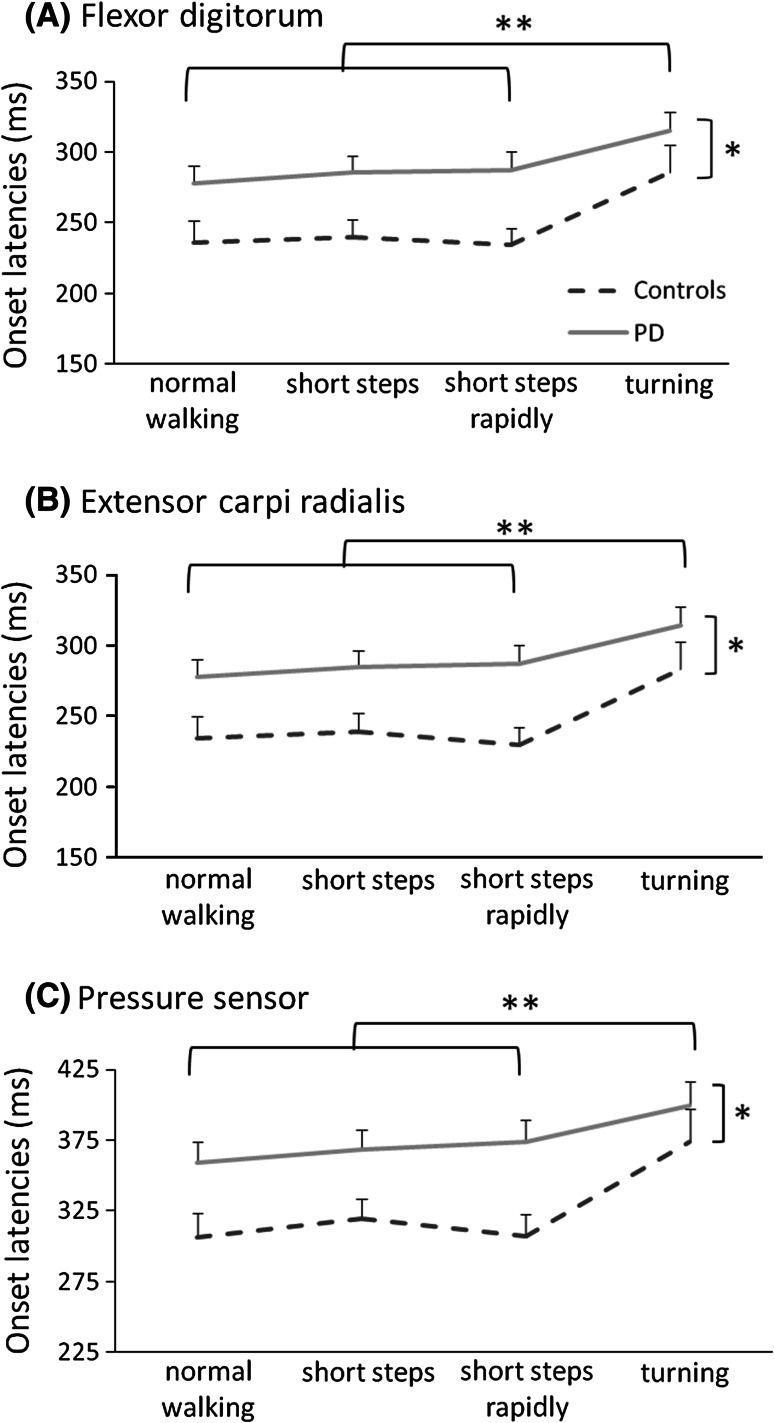
Onset latencies (+SE) for PD patients (*solid grey lines*) and controls (*dashed black lines*) for each walking condition in **a** flexor digitorum; **b** flexor carpi radialis and **c** pressure sensor. *Significant main effect of group; **significant difference between turning and all other conditions

All participants were able to complete the measurement without stopping during the gait tasks. When squeezing the ball, the flexor digitorum and extensor carpi radialis muscle were near-simultaneously activated. Onset latencies of the flexor digitorum muscle were delayed by on average 42 ms in PD patients (291 ± 68 ms) compared to controls (249 ± 55 ms; group; *F*_1,41_ = 5.154, *p* = 0.029, see Fig. 1a), but this delay did not differ between tasks (task × group*; F*_3,41_ = 0.762, *p* = 0.517). In addition, the analysis yielded a significant main effect of task (*F*_3,41_ = 12.068, *p* < 0.001). Post-hoc paired samples *t* tests showed that in both PD patients and controls, reaction times were on average 37 ms longer during the turning condition compared to the other tasks [*t*(42) < −4.085, *p* < 0.001].

For the turning condition, we also verified whether reaction times differed between turns towards the most and the least affected side in PD patients with marked motor asymmetry. Motor asymmetry was defined as ≥30 % difference between left and right sided items of the MDS-UPDRS motor sub-section. *N* = 18 PD (62.1 %) patients had marked motor asymmetry. For these patients, average RTs were 290 ± 77 ms for turns towards the most affected side versus 313 ± 85 ms for turns towards the least affected side, which difference was not significant (paired *t* test, *p* = 0.233).

The results for onset latencies in extensor carpi radialis and for movement onsets using the sensor located within the ball (Fig. 1b, c) yielded an equivalent pattern of statistical significance and are therefore not described in detail here.

### Reaction times during FOG-episodes

During the measurement, FOG was observed in nine patients and we managed to record reaction times to the auditory stimulus both when freezing and non-freezing in all these patients. Eight of them froze during the turning condition (*n* = 37 FOG-episodes). Two patients froze when walking with short steps (*n* = 10 FOG-episodes), and five when walking with short steps rapidly (*n* = 24 FOG-episodes). FOG was not observed during the normal walking task. There was 100 % agreement between the raters on the presence or absence of FOG when scoring the videos.

**Fig. 2:**
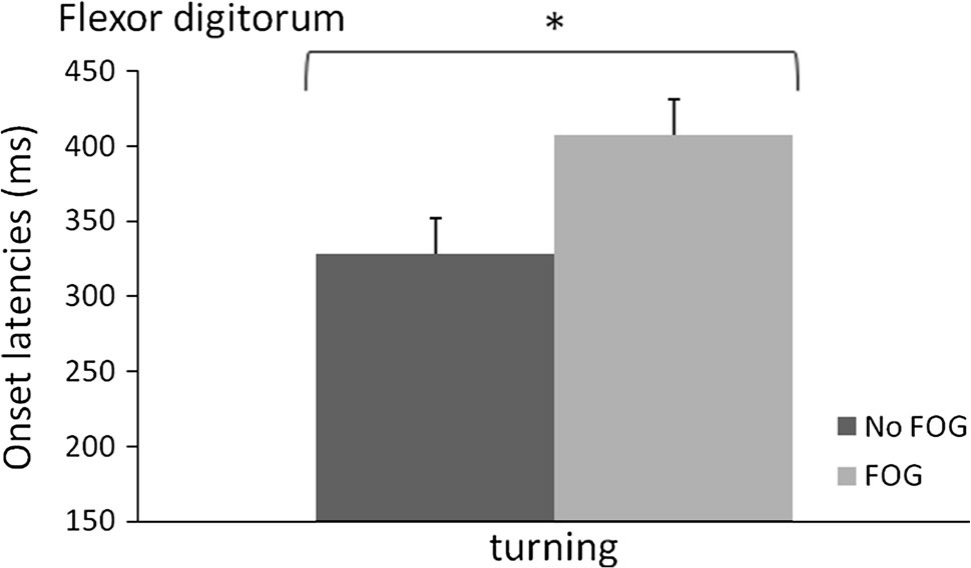
Onset latencies (+SE) in turning trials without FOG-episodes (*dark grey*) and with FOG-episodes (*light grey*). *Significant difference

For patients with FOG during the turning condition, we compared reaction times in flexor digitorum during a FOG-episode to those in turning trials without FOG. Reaction times were on average 79 ms slower during a FOG-episode (407 ± 62 ms) compared to turning trials without FOG [328 ± 65 ms; *t*(6) = 3.101, *p* = 0.02] (see Fig. 2), whereas the non-freezing reaction times in these participants were not different from PD patients that did not freeze at all during turning [310 ± 77 ms; *t*(27) = −0.592, *p* = 0.50]. Again, these results were mirrored in extensor carpi radialis and movement onset latencies, which statistics are therefore not further reported. We observed the same pattern during walking with short steps rapidly. Reaction times were on average 57 ms longer during FOG-episodes (327 ± 64.3 ms) compared to trials without FOG (270 ± 73 ms), but this analysis included only five people and the difference did not reach significance [*t*(4) = 1.69, *p* = 0.17].

## Discussion

In the present study we evaluated manual reaction times in PD patients and healthy controls during walking, walking with short steps, walking with short steps as rapidly as possible and full rapid turns. We observed an overall delay in reaction times in PD patients compared to controls, regardless of the walking condition. In both groups, reaction times were significantly longer during turning compared to the other conditions; in the PD group, the delay in reaction times during turning was independent of turning direction (i.e. towards most or least affected side). In the conditions that yielded substantial numbers of FOG-episodes in our group of PD patients (walking with short steps rapidly and turning), reaction times were delayed during FOG-episodes compared to trials without FOG, albeit only significantly for the turning condition.

Previous studies indicated that walking is not merely an automatic task, but also relies on executive functions and attention (Sparrow et al. 2002; Bloem et al. 2006). Our observation that reaction times were longer during turning compared to the other tasks shows that this challenging gait task indeed required more attentional resources compared to the other walking tasks. For the other two challenging gait tasks (i.e. walking with short steps and walking with short steps rapidly), however, no reaction time delays were observed. This finding is somewhat unexpected, in light of the postulated role of attentional compensation for PD-related gait deficits. Aggravation of these gait deficits and the occurrence of FOG was suggested to be due to the greater attentional and executive demands involved in challenging walking tasks (Giladi et al. 2006; Vandenbossche et al. 2013). We indeed observed substantial numbers of FOG-episodes (*n* = 34) during the two tasks involving walking with short steps, which confirms their reported ability to provoke freezing. We did not find evidence, however, for these tasks to impose major additional cognitive demands compared to normal walking.

People with PD who perform a secondary task during walking often show a performance decrement in one or both of the tasks (Fuller et al. 2013). Most studies focused on motor performance, showing greater decrements in spatiotemporal gait parameters under dual task conditions in PD patients compared to healthy controls (Bloem et al. 2006; Hausdorff et al. 2003). In contrast, secondary task performance was reported in relatively few studies (Spildooren et al. 2010; Rochester et al. 2014; O’Shea et al. 2002; Kelly et al. 2014). Our finding of an overall delay in reaction times in PD patients compared to controls is in agreement with these previous studies, which demonstrated significantly worse performance on a secondary cognitive task in PD patients compared to controls during walking (Kelly et al. 2014), and even more pronounced delays in PD patients with FOG during a turning task (Spildooren et al. 2010). These results indicate that difficulties in performing a secondary task in PD patients may be due to the utilization of attentional resources to compensate for their gait impairments (Willems et al. 2007; Browner et al. 2010; Peterson et al. 2012).

The present results, however, do not support our hypothesis of a disproportionate increase of attentional strategies in PD patients during more challenging gait tasks, as the delay in reaction times during turning (not including reaction times during freezing episodes) was similar between patients and healthy controls. This finding contrasts with the results of a study that evaluated a cognitive dual task during turning, and that found no decrements in secondary cognitive task performance during full turns in both PD patients without FOG and in controls (Spildooren et al. 2010). The presently observed delayed reaction times might be related to the instruction to perform the turns as rapidly as possible, which constitutes a more challenging task compared to turning at a comfortable pace (Snijders et al. 2012). Indeed, the nature of the specific instructions given to participants has a relevant influence on the performance of both the primary task at hand, as well as the secondary task(s) (Bloem et al. 2006).

The challenging nature of the turning task was also exemplified by the more frequent occurrences of FOG compared to the other tasks, which is in line with previous reports (Snijders et al. 2012; Schaafsma et al. 2003). Interestingly, the within-subjects analysis demonstrated that reaction times were delayed during FOG-episodes compared to trials without FOG, whereas non-freezing reaction times in these participants were similar to those of the PD patients who did not freeze during turning. It suggests that the freezers did not allocate greater attentional resources to the turning task itself, but rather used additional resources to overcome the FOG-episodes evoked by turning. This finding supports the suggested utilization of a neural circuitry engaged in attention to overcome FOG-episodes (Browner et al. 2010).

Our discrete secondary cognitive task was very sensitive in detecting between-group and between-task differences in reaction times, and it had the additional advantage of flexibility in administering stimuli during both freezing and non-freezing episodes. However, it may be argued that this task does not optimally represent the typical impairments in executive functioning that have been associated with PD-related gait impairments (Smulders et al. 2013) and FOG (Amboni et al. 2008; Smulders et al. 2012). Several studies on dual task interference during walking used an auditory Stroop task (Kelly et al. 2014; Smulders et al. 2012), which may better reflect executive functioning, and can also be administered as a discrete stimulus. This task, however, requires many more trials for reliable estimates of performance because it involves congruent as well as incongruent stimulus–response sets. Moreover, reaction times can be confounded by changes in response accuracy. Also, the auditory Stroop task has much longer stimulus–response intervals (a factor 3–4 times greater than those for our ball-squeezing task) (Smulders et al. 2012). These disadvantages arguably render the auditory Stroop task less suitable for application during freezing episodes, as these are typically too brief and infrequent to allow for full evaluation of secondary task performance. Hence, we feel that our simple-reaction ball-squeezing task was appropriate for the purpose of this study. Yet, it would be of interest to determine whether a choice reaction task (e.g. squeezing with left or right hand in response to distinct auditory stimuli) may be even more sensitive in revealing subtle differences between individual tasks, as selecting the appropriate response to a specific stimulus requires more cognitive processing than the singular stimulus–response mapping in a simple reaction task.

A limitation of our study is the lack of a ‘baseline’ condition that involved responding to the auditory stimulus in a stationary position (sitting or standing). Therefore, we cannot exclude the possibility that the PD-related delays in reaction times were due to general slowness, rather than being related to interference of gait with the cognitive task (Rochester et al. 2014; Amboni et al. 2013). Recent studies, however, found no differences between PD patients and controls in simple reaction times while subjects sat or stood in a stationary position (Rochester et al. 2014; Nonnekes et al. 2014; Fernandez-Del-Olmo et al. 2013) suggesting that the presently observed delay in reaction times was indeed caused by interference between gait and secondary tasks. Hence, our results appear consistent with the notion that PD patients have difficulties performing a secondary cognitive task while walking (Giladi and Hausdorff 2006; Willems et al. 2007).

In conclusion, our results suggest that turning requires more attentional resources compared to other walking tasks, both in PD patients and in healthy controls. The observation of delayed reaction times during FOG-episodes compared to trials without FOG suggests utilization of additional cognitive resources to overcome FOG-episodes.
